# Can Visual Acuity Be Reliably Measured at Home? Validation of Telemedicine Remote Computerised Visual Acuity Measurements

**DOI:** 10.22599/bioj.179

**Published:** 2021-06-16

**Authors:** Ailsa Ritchie, Silva Atamian, Nilpa Shah, Alistair Laidlaw, Christopher Hammond

**Affiliations:** 1 Guy’s and St Thomas’ NHSFT, GB; 2 King’s College London, GB

**Keywords:** Telemedicine, visual acuity, vision testing, amblyopia, refractive error

## Abstract

**Purpose:**

The recent pandemic has identified the need for telemedicine assessment of ophthalmology patients. A vital component of such assessment is visual acuity (VA) measurement. The aim of this study was to determine the feasibility and reliability of computerised ‘at home’ VA measurements using COMPlog software.

**Methods:**

A Bland Altman method comparison study of worse eye ‘in clinic’ and ‘at home’ orthoptist-supervised COMPlog computerised VA measurements. Subjects underwent gold standard semi-automated computerised test and retest logMAR VA measurements on their habitually corrected worse eye both ‘in clinic’ and ‘at home.’ The orthoptist ran the test from the eye clinic with the patient viewing a secondary PC monitor either in the same clinic room or at home. A screen sharing voice and video conferencing application and standard consumer IT hardware were employed to present the test optotypes in the patient’s home.

**Results:**

23 paediatric and 13 adult patients with a range of ocular diseases and worse eye visual acuities were included (range –0.14 to 1.06 logMAR). No significant bias was found between ‘in clinic’ and ‘at home’ measurements (mean –0.01 logMAR and 95% confidence interval –0.03, 0.00 logMAR). Test-retest variability of ‘in clinic,’ ‘at home’ and ‘in clinic’ versus ‘at home’ measurements were within normal reported ranges at 0.12 logMAR (6 ETDRS letters) or less.

**Conclusion:**

Remote home VA testing performed by an eye care professional using a semi-automated VA measurement program and video conferencing application provided unbiased measurements with acceptable test-retest reliability. Home testing was both feasible and acceptably reliable in appropriately equipped patients.

## Introduction

Accurate visual acuity (VA) measurements are fundamental to ophthalmology assessment and clinical decision making. In a clinic setting, patients routinely have their VA measured by a trained healthcare professional ideally with a validated logarithmically scaled chart or computerised test. The gold standard in this regard being single letter scoring measurements taken using Early Treatment Diabetic Retinopathy Study (ETDRS) charts (Ferris et al. 1986; Beck et al. 2003).

During the COVID-19 pandemic, routine ophthalmology outpatient activity has been reduced across England (Gardner, Fraser & Peytrignet 2020). This followed guidelines from the Royal College of Ophthalmologists (RCOphth 2020). In addition to this, many patients have chosen not to come to hospital for a face-to-face appointment thereby delaying care in order to minimise exposure to the virus (Kalra et al. 2020). This has led to an increase in virtual consultations being used to triage and manage ophthalmology patients remotely (Gerbutavicius et al. 2020).

There are long term advantages of remote, real-time consultations compared to face-to-face clinic consultations, which include reduced travel costs and travel time for the patient and their families and keeping patients in a familiar environment (Greenhalgh et al. 2016; Kalra et al. 2020). Over the last decade there has been an increasing global investment in telemedicine technologies. This has resulted in a number of platforms which are Health Insurance Portability and Accountability Act (HIPAA) compliant, meaning that clinicians and patients can have video consultations over an internet connection that is secure and protected against data breaches (HIPAA Journal 2020). The potential for digital health to widen or narrow health inequalities is unknown and (Rich, Miah & Lewis 2019) caution against an uncritical adoption of digital health solutions. In ophthalmology, video consultations are useful for taking a history and performing an external eye examination. Further clinical investigations require additional software, such as online applications for VA testing, or additional instruments for imaging or functional assessments, such as optical coherence topography, corneal topography and perimetry.

In a recent review, 42 online or mobile applications (apps) for self-assessment VA tests were identified (Yeung et al. 2019). All of these require downloading onto a personal device and some require payment. The validity and reliability of vision testing using apps by non-healthcare professionals in the home setting has not been established (The Royal College of Ophthalmologists and the British and Irish Orthoptic Society 2020). Even with clear instructions, an unwitnessed VA test carried out by a patient, parent or guardian at home is at risk of being inaccurate due to a number of uncontrolled variables. These include uncorrected changes in viewing distance, unnoticed peeking through occlusion, terminating the test too early or giving clues to the patient and using incorrect glasses. The Royal College of Ophthalmologists and the British & Irish Orthoptic Society have jointly recommended a cautious approach to the use of such applications in monitoring vision in children, and have recommended their use only under the guidance of a trained healthcare professional (The Royal College of Ophthalmologists and the British and Irish Orthoptic Society 2020).

COMPlog is a commercially available validated PC based semi-automated computerised logMAR VA measurement system (COMPlog Computerised Clinical Vision Measurement Systems Ltd London UK). It was developed, validated and is routinely used at St Thomas’ Hospital, London, UK, (Laidlaw et al. 2008; Shah et al. 2010, 2012; Bokinni et al. 2015). The test is run by a health care professional with the patient viewing and identifying size calibrated single or linear optotypes presented on a secondary monitor. COMPlog measurements have been shown in method comparison studies on a like for like basis to be unbiased and of similar test-retest variability (TRV) to gold standard ETDRS chart single letter scoring measurements (Laidlaw et al. 2008). The validity of this program for remote vision testing in 50 adults has been independently determined (Srinivasan et al. 2012). In this study, volunteer university students and staff were placed in a clinic room set up for COMPlog vision testing and they communicated with an optometrist via a telephone call to have their visual acuity tested remotely. To our knowledge, COMPlog has not been used to test a patient’s visual acuity at home remotely. It is possible to present size calibrated images of the COMPlog secondary monitor both in clinic and at home via video conferencing screen sharing applications, which also allow video observation of the patient undergoing the test by the eye care professional and voice interaction. Combining a screen sharing video conferencing application and the COMPlog acuity measurement program in this way potentially facilitates measurement of gold standard ‘at home’ VA.

Our aim was to determine the feasibility and reliability of ‘at home’ acuity measurements. Reliability was determined in terms of bias and TRV of ‘at home,’ ‘in clinic’ and ‘at home’ versus ‘in clinic’ measurements. Index of Multiple Deprivation scores were calculated and used to evaluate the impact of socioeconomic status on the execution of an ‘at home’ test.

## Methods

Inclusion criteria were consenting consecutive patients with corrected worse eye stable vision between –0.2 and 1.2 logMAR who were attending an ophthalmology clinic for a face to face follow up appointment. Participants had to own a desktop, laptop or tablet web browser device with a front facing camera and have domestic internet access.

Recruited patients underwent standardised test and retest measurements of the acuity of their worse seeing eye both ‘in clinic’ and ‘at home’ within one week of each other. An initial cohort of patients had ‘in clinic’ VA testing followed by ‘at home’ VA testing and then the order was reversed. Habitual correction was used for both ‘in clinic’ and ‘at home’ VA measurements with the fellow eye occluded. All clinic and remote vision tests were carried out by the same orthoptist (SA).

The index of multiple deprivation (IMD) 2019 was calculated for each individual using the online postcode look up tool produced by the UK Ministry of Housing, Communities and Local Government (UK Ministry of Housing, Communities and Local Government 2019). This IMD combines measures of income, employment, education, health, crime, access to housing and services, and the living environment in order to create an overall score of multiple deprivation (Mclennan et al. 2019). Scores for small areas throughout England are ranked and presented as deciles. Decile 1 represents the most deprived 10% of neighbourhoods and decile 10 represents the least deprived 10% of neighbourhoods in England.

This project was approved by our institution’s audit and quality improvement project team. Data collection adhered to the tenets of the Declaration of Helsinki and the UK Data Protection Act.

### Calibration

Prior to each test session (both ‘in clinic’ and ‘at home’), the COMPlog software was calibrated in order to ensure that letter sizes were displayed accurately for each logMAR size. This was done by physically measuring the size of a calibration cross presented on the secondary monitor, either by the orthoptist in clinic or by the patient or parent at home during remote testing, supervised by the orthoptist.

### COMPlog visual acuity testing

The same COMPlog Thresholding measurement algorithm was employed both ‘in clinic’ and ‘at home’ to measure VA. COMPlog Thresholding consists of two phases ‘range finding’ and ‘thresholding.’ In range finding a single crowded Sheridan Gardiner letter is presented in ascending or descending 0.2 logMAR steps from 0.8 logMAR until the smallest recognised size is identified. Thresholding commences 0.2 logMAR larger than this optotype size. In this study thresholding consisted of five crowded Sheridan Gardiner letters presented per line. Optotypes were spaced half a letter width (2.5 stroke widths) apart (Shah et al. 2010). Lines of letters were surrounded by a crowding box of one stroke width separated by 2.5 stroke widths from the letter borders. The response to each individual letter presented was recorded by the orthoptist as ‘correct’ or ‘incorrect’ on the COMPlog software. The test continues with sequentially smaller lines of letters in 0.1 logMAR steps until all five letters on one line have been incorrectly identified. In the event that errors are made on the first presented line, sequentially larger line sizes are presented until all five responses on a line are correct with thresholding descending from that size. Depending on screen calibration factors larger lines are broken up into single, pairs or triplets of crowded letters, but five letters are presented and scored at each line size. The test terminates when the pre-defined failure criteria, in this case five letters wrong, have been met. Automated single letter scoring giving credit for each correctly identified letter is performed and presented. The ‘refresh’ option was used to show a patient a second presentation at the same stimulus level if an attention lapse was suspected. VA was recorded in 0.02 decimal logMAR units. No finger pointing to the letters was used in clinic or with remote vision testing. The VA of each eye was tested twice both in clinic and at home (i.e. four tests) to assess test-retest variability.

### Clinic visual acuity testing

Clinic rooms were set up with the patient seated three metres away from a wall mounted secondary monitor, the test being controlled by the orthoptist from the primary monitor of the PC. Normal room illumination was used and windows were curtained to avoid screen glare.

### Remote visual acuity testing

Patients received a patient information leaflet on home vision testing, which explained the requirements and gave full instructions on room set up. Requirements included a personal computer, laptop or tablet with a forward-facing camera and a reliable internet connection, a ruler for screen calibration and enough space to have three metres between a chair and the screen of the device. Patients were given a three metre length of string or they used their own tape measure. A video consultation was set up using the Attend Anywhere platform. Patients were asked to set the screen brightness on their device to 100% brightness. Curtains or blinds were closed and the screen positioned to avoid reflections and/or glare. Room lights were turned on to full brightness to simulate clinic light levels as closely as possible. The distance between the screen and the patient’s eye level when sitting on a chair three metres from their device was checked by the patient, observed by the orthoptist. The screen share function was used to display the COMPlog secondary monitor on the patient’s device. The COMPlog software was controlled by the orthoptist (SA) who observed the patient’s fellow eye occlusion and movement towards the screen during VA testing and talked to the patient and heard their responses throughout the video consultation.

Minimum System requirements to run the Attend Anywhere application were Microsoft Windows 7, iOS 13 or Android 5.1. Minimum browser requirements were Chrome 80 or later, Safari 12.4 or later or Edge 83 or later. The recommended bandwidth download and upload speeds were 1.1 and 0.7 Megabites per second respectively (Attend Anywhere 2020).

### Statistical analysis

Tests of normality were performed on each data set. The methods of Bland-Altman were used to quantify bias (mean and 95% confidence interval of the mean) between home and clinic vision testing as well as TRV expressed as 95% confidence limits of agreement (mean ± 2SD) for paired home and clinic COMPlog algorithm scores (Bland & Altman 2010).

## Results

Thirty-nine patients were invited to take part and three (7%) of these patients were excluded. These three patients were aged 11, 54 and 65 years old and they could not establish an Attend Anywhere session as their personal devices did not meet the system or browser requirements for Attend Anywhere.

Thirty-six patients underwent home and clinic vision testing within a seven-day time period. All patients had had VA testing in clinic previously. Patient characteristics are shown in ***Table 1***.

**Table 1:** Patient characteristics. SD = standard deviation IQR = interquartile range IMD = index of multiple deprivation.


	CHILDREN (UNDER 16 YEARS OLD)	ADULTS (OVER 16 YEARS OLD)	ALL

Number of patients	23 (64%)	13 (36%)	36 (100%)

Sex	15M 8F	7M 6F	22 M (61%) 14 F (39%)

Age (years) Mean (SD), range	9 (3), 5–15	49 (14), 26–75	23 (21), 5–75

The English IMD 2019 decile (mean, range)	6, 2–10	5, 2–10	6, 2–10

Worse eye habitually corrected visual acuity (1^st^ clinic VA test)	Median 0.12 IQR 0.01–0.25 Range –0.04 to 0.6	Median 0.04 IQR –0.02–0.18 Range –0.06 to 1.06	Median 0.1 IQR 0.02–0.245 Range –0.1 to 1.06

Ocular pathology in the worse eye:			

No ocular pathology (Inc. refractive error and JIA with no ocular sequelae)	11	1	12

Strabismus	2	8	10

Amblyopia	7	2	9

Other pathology (Inc. cataract, retinal pathology, optic nerve pathology)	3	2	5


Twenty-two patients (58%) had a clinic VA test followed by a home VA test and 14 patients (42%) had a home VA test followed by a clinic VA test. Twenty clinic tests were timed. Overall, the mean test time was five minutes (mins) (range 2–8 mins), with a mean test time of five minutes for children (n = 8 range 3–8mins) and five minutes for adults (n = 12 range 2–8 mins). Twenty-three remote vision tests were timed after room set up and calibration. Overall, the mean test time was six mins (range 2–14 mins), with a mean test time of seven mins for children (n = 11 range 3–14 mins) and a mean test time of five mins for adults (n = 12 range 2–9 mins).

The device used for home vision testing was a laptop or desktop computer for 27 patients (75%) and a tablet in nine patients (25%). The use of tablets for home VA testing was slightly higher for children (30%) compared to adults (15%).

**Figure 1:**
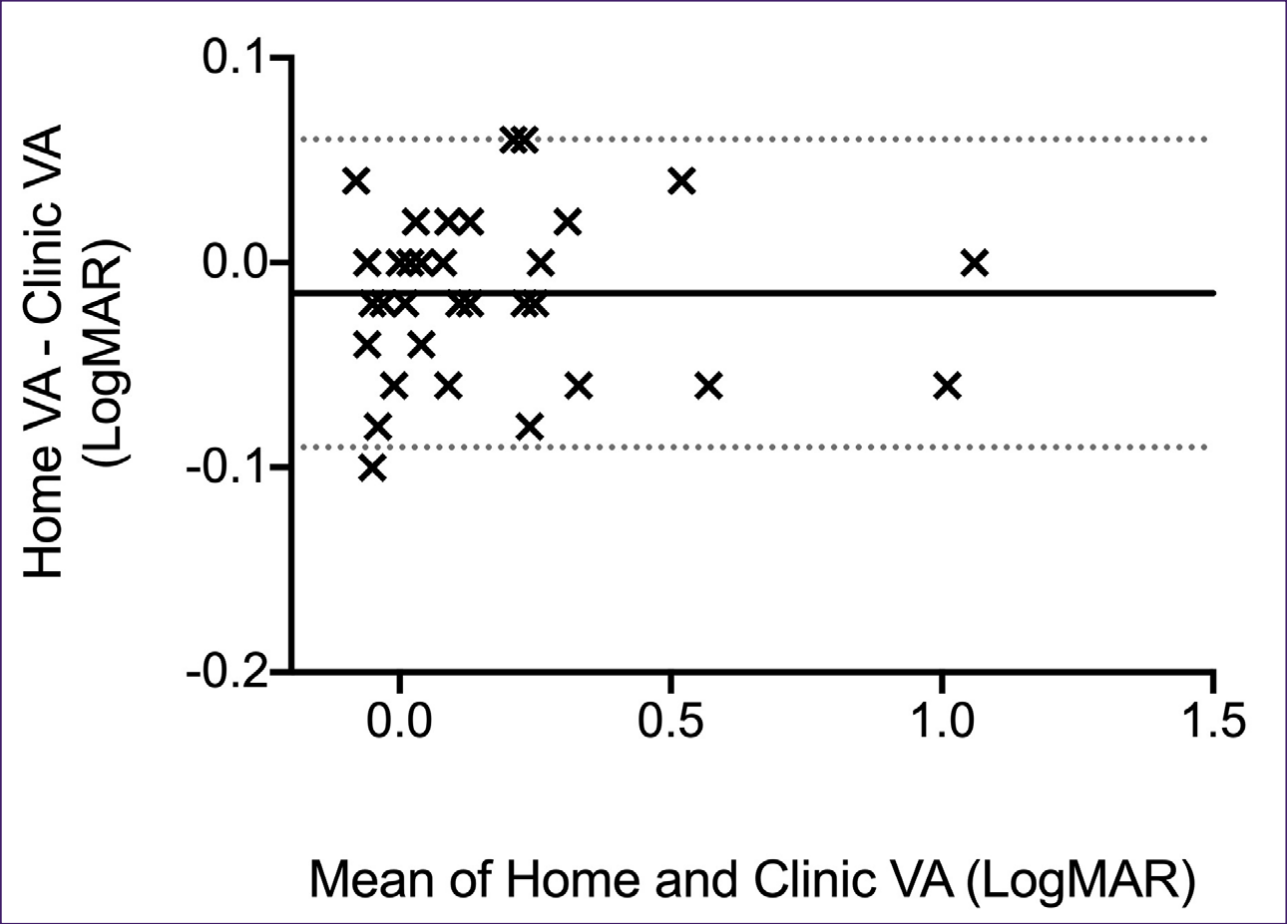
A Bland-Altman comparison of ‘at home’ and ‘in clinic’ paired VA measurements. Solid line: mean difference in logMAR VA between ‘at home’ and ‘in clinic’ VA measurements. Area within the dotted lines: 95% limits of agreement between logMAR VA ‘at home’ and ‘in clinic.’

The differences in paired tests in each data set conformed reasonably to a normal distribution allowing Bland-Altman analysis and description. There was no systematic difference between the acuity being measured ‘at home’ compared to ‘in clinic,’ nor any obvious proportional bias, as shown in ***Figure 1*** and ***Table 2***.

**Table 2:** Comparison of ‘at home’ and ‘in clinic’ VA testing.


	WORSE EYE 1^ST^ ‘IN CLINIC’ AND ‘AT HOME’ TEST VARIABILITY (logMAR)	WORSE EYE ‘IN CLINIC’ TEST-RETEST VARIABILITY (logMAR)	WORSE EYE ‘AT HOME’ TEST-RETEST VARIABILITY (logMAR)

Mean Difference	–0.01	0.01	0.00

95% Confidence Interval Mean	–0.03, 0.00	–0.01, 0.01	–0.02, 0.02

Standard deviation	0.04	0.03	0.06

95% Confidence limits of agreement	+/–0.08	+/–0.06	+/–0.12


The variability of ‘at home’ and ‘in clinic’ VA measurements, indexed by the 95% confidence limits of agreement and shown in ***Table 2***, were slightly greater at home compared to in clinic (0.12 cf 0.06 logMAR).

Subgroup analysis of the 23 children showed no significant bias between ‘at home’ and ‘in clinic’ measurements (mean difference –0.02 logMAR, 95% CI –0.03 to –0.00 logMAR) and TRV was +/–0.11 logMAR for home VA testing. These are similar to the results of adults and children combined. Analysis of 18 patients who were from more deprived areas, in deciles 1–5 of the IMD 2019, showed no significant bias between ‘at home’ and ‘in clinic’ measurements (mean difference –0.02 logMAR, 95% CI –0.04 to –0.00 logMAR). The TRV was +/–0.16 and +/–0.06 logMAR for home and clinic VA testing respectively. The differences in home vision test results were similar to the differences in clinic test results in 18 patients from less deprived areas, in deciles 6–10 of the IMD 2019, with a TRV of +/–0.07 and +/–0.06 logMAR for home and clinic VA testing respectively.

## Discussion

Our results suggest using COMPlog screen share through the Attend Anywhere platform allows VA to be measured ‘at home’ with the same high degree of rigor as occurs in a clinic. The ‘at home’ results were unbiased compared to clinic measurements and showed low TRV. Clinically, this means that ‘in clinic’ and ‘at home’ VA test results can be directly compared, and progress monitored. It is generally accepted that individuals with stable vision change by less than two lines of logMAR VA measurements on repeat testing (Rosser et al. 2003) TRV, as indexed by 95% limits of agreement, have previously been reported between ±0.07 logMAR and ±0.20 logMAR (Elliott & Sheridan 1988; Rabbetts 1989; Reeves, Wood & Hill 1993; Vanden Bosch & Wall 1997; McMonnies, Hazel and Elliott 2003; Rosser et al. 2003). The limits of agreement in the TRV for overall ‘in clinic’ and ‘at home’ VA testing presented here are both well within this difference. When the capacity to see patients face to face in clinic is limited, this is a useful adjunct to a remote video consultation.

A range of personal devices were used by patients, with differing screen sizes. The Attend Anywhere window has a standard landscape ratio of 16:9 and fills two thirds of the device screen when maximised during a video consultation. It is important that patients do not alter the screen zoom after calibration. For larger optotypes displayed on the second screen, which is the patient’s device screen during home VA testing, the letter lines are broken up into single, pairs or triplets of letters, so that they fit on the display screen. Five letters are always presented and scored at each line size. ***Table 3*** shows the maximum logMAR VA that can be tested remotely with various personal device screen sizes. The calibration process means that the eye care professional is able to see the secondary monitor image as presented in the video conference software window on the patient’s screen. They can therefore detect when letters or lines exceed the available screen size.

**Table 3:** Screen size ‘at home’ requirements for remote crowded VA testing.


SCREEN SIZE	MAXIMUM CROWDED logMAR VA THAT CAN BE TESTED AT 3m	EXAMPLE DEVICE

24”	1.40	Desktop screen

15”	1.10	Laptop

10”	0.9	Tablet

6”	0.4	Smartphone


Screen brightness on personal devices was not standardised, nor were the light levels in patient’s homes, both of which have been shown to have a small impact on VA testing (Bhorade et al. 2013). During the set up all devices were set to their maximum brightness. Room light levels were also observed during the set up and optimised as much as possible. Although uncontrolled, this does reflect real life home vision testing rather than remote vision testing in a clinic setting which has previously been reported (Srinivasan et al. 2012). The lack of bias between datasets suggests that such presentation factors are not significantly affecting test performance.

This method of home vision testing is only available to patients with a suitable device and internet access. Three patients who volunteered to have ‘at home’ VA testing were unable to login to the Attend Anywhere consultation software. The majority of patients, who come from a broad range of socio-economic areas as measured by the Index of Multiple Deprivation 2019, owned a suitable personal device. The Office of National Statistics reported 96% of households in Great Britain had internet access between January and February 2020 (Office for National Statistics 2020). These findings are therefore likely to be applicable to a large proportion of the UK population. Low-cost web browser devices could conceivably be issued or loaned as an alternative to requiring hospital attendance in patients who are not suitably equipped.

Patients from more deprived areas had a slightly higher TRV at home compared to in clinic (0.16 cf 0.06 logMAR). This difference was not seen in patients from less deprived areas. Clinic rooms are set up for examinations and it may be harder for patients from more deprived areas to find a similar space to do vision testing remotely at home than for patients from less deprived areas. However, a TRV of ±0.16 logMAR is still within the reported range of previous studies (Rosser et al. 2003) so should not preclude any patients from home vision testing.

Screen freezes were a temporary problem during some ‘at home’ VA tests, resulting in a slightly longer test time, but no tests were abandoned due to the quality of the video consultation once the consultation had started. The mean test times were comparable for home and clinic VA tests but were slightly longer in children during home vision testing than in clinic. Additional time for set up and calibration was also needed for home vision tests, which was not recorded.

One experienced orthoptist in a single centre performed all the VA measurements in clinic and remotely, which eliminated inter-tester variability. They were not blinded to the previous VA test results, but care was taken not to see the most recent VA test result before the start of the second home or clinic VA test. The semi-automated forced choice algorithm is designed to reduce the effect of observer bias.

This was a first in class method comparison study of ‘at home’ versus ‘in clinic’ testing in which no systematic bias was found. The numbers are typical of a VA test method comparison study. All participants had received an in-clinic VA assessment prior to the ‘in clinic’ and ‘at home’ vision tests presented here and these findings may or may not be applicable to ‘at home’ assessment without prior experience. We did not include young children requiring VA testing with picture optotypes, although this is possible using the same set up. We only tested this one combination of acuity testing and virtual consultation software, and so these results may not be generalisable to other systems. Further research on a larger number of patients would be needed to examine inter-tester variability, and the effect of a wider range of age and pathologies.

In summary we have shown that unbiased ‘at home’ VA measurements of comparable precision and reliability to gold standard ‘in clinic’ measurements may be made through the combined use of a validated semi-automated VA measurement program and a video conferencing application with appropriate supervision by an eye care professional.
